# Enrichment and Analysis of Stable 1,4-dioxane-Degrading Microbial Consortia Consisting of Novel Dioxane-Degraders

**DOI:** 10.3390/microorganisms8010050

**Published:** 2019-12-25

**Authors:** Tanmoy Roy Tusher, Takuya Shimizu, Chihiro Inoue, Mei-Fang Chien

**Affiliations:** Graduate School of Environmental Studies, Tohoku University, 6-6-20 Aoba, Aramaki, Aoba-ku, Sendai 980-8579, Japan; trtusher.esrm@gmail.com (T.R.T.); takuya.shimizu.p3@dc.tohoku.ac.jp (T.S.); chihiro.inoue.b1@tohoku.ac.jp (C.I.)

**Keywords:** 1,4-dioxane, biodegradation, microbial consortia, dioxane-degrader, *Variovorax*

## Abstract

Biodegradation of 1,4-dioxane, a water contaminant of emerging concern, has drawn substantial attention over the last two decades. A number of dioxane-degraders have been identified, though many of them are unable to metabolically utilize 1,4-dioxane. Moreover, it is considered more preferable to use microbial consortia rather than the pure strains, especially in conventional bioreactors for industrial wastewater treatment. In the present study, a stable 1,4-dioxane-degrading microbial consortium was enriched, namely 112, from industrial wastewater by nitrate mineral salt medium (NMSM). The consortium 112 is capable of utilizing 1,4-dioxane as a sole carbon and energy source, and can completely degrade 1,4-dioxane up to 100 mg/L. From the consortium 112, two 1,4-dioxane-degrading bacterial strains were isolated and identified, in which the *Variovorax* sp. TS13 was found to be a novel 1,4-dioxane-degrader that can utilize 100 mg/L of 1,4-dioxane. The efficacy of the consortium 112 was increased significantly when we cultured the consortium with mineral salt medium (MSM). The new consortium, N112, could utilize 1,4-dioxane at a rate of 1.67 mg/L·h. The results of the ribosomal RNA intergenic spacer analysis (RISA) depicted that changes in the microbial community structure of consortium 112 was the reason behind the improved degradation efficiency of consortium N112, which was exhibited as a stable and effective microbial consortium with a high potential for bioremediation of the dioxane-impacted sites and contaminated industrial wastewater.

## 1. Introduction

1,4-dioxane (C_4_H_8_O_2_), a colorless and flammable heterocyclic ether compound, has appeared as an emerging surface water and groundwater contaminant [1,2,3]. The International Agency for Research on Cancer (IARC) and the U.S. Environmental Protection Agency (USEPA) have classified 1,4-dioxane as a Group 2B and B2 agent, respectively, which implies that it has a high potential for causing cancer in humans [4,5,6]. This xenobiotic compound has also been listed as a priority pollutant by USEPA [2,6] and as a superfund priority substance by the Agency for Toxic Substances and Disease Registry (ATSDR) due to its probable toxic effects, high chemical stability, persistence in the natural environment, and potency to contaminate drinking water [7]. 1,4-dioxane is widely used in various industries as a wetting and dispersing agent [8] and as a solvent for the manufacturing of organic chemicals used for industrial production [3,6,9]. It is also used as a surfactant in foods, detergents, and cosmetics, and particularly as a stabilizing agent for chlorinated solvents, especially 1,1,1-trichloroethane (1,1,1-TCA), trichloroethene (TCE), and tetrachloroethene (PCE) [8,10]. Moreover, it is formed as an undesirable by-product during the manufacture of ethylene oxide, ethylene glycol, polyesters, and surfactants, including polysorbates, sorbitol, and sorbitan [8,9,11]. Due to the extensive use and unintentional formation of 1,4-dioxane during industrial processes, this xenobiotic compound is often found in domestic sewage and industrial wastewater at a concentration of several hundred mg/L [12,13].

Due to its unique physicochemical properties like extreme hydrophilicity (Log K_ow_: −0.27), low hydrolyzability, less volatility (Henry’s law constant: 4.80 × 10^−6^ atm-m^3^/mol at 25 °C), low absorbability to solids (Log K_oc_: 1.23), and less biodegradability (<5% in 60 days under aerobic condition in water) [2,5,13,14], most conventional physicochemical and biological treatment technologies (e.g., air stripping, thermal desorption, activated carbon adsorption, membrane filtration, chemical coagulation, and activated sludge treatment) are found to be ineffective to remove 1,4-dioxane from the contaminated water or wastewater [3,6,8,13]. As a result, industrial discharge of 1,4-dioxane contaminated wastewater, even after treatment using conventional physicochemical and biological technologies, can contaminate the surface water and groundwater with 1,4-dioxane [9]. Advanced oxidation processes (AOPs), such as hydrogen peroxide (H_2_O_2_) treatment with ozone (O_3_) or with ultraviolet (UV) light, can effectively degrade 1,4-dioxane [3,15,16] and, thus, most of the ongoing remediation activities at 1,4-dioxane contaminated sites depend on treatment strategies involving AOPs [17,18]. However, the AOPs have several shortcomings, such as the technology is expensive and energy-intensive, requires high amount of chemicals, produces harmful by-products which demand further biological treatments, and is inefficient in removing 1,4-dioxane when co-contaminants or additional carbon sources are present [3,12,13]. These disadvantages limit the feasibility of the application of AOPs for in-situ bioremediation of large 1,4-dioxane contaminated sites. As a result, the biological treatment of 1,4-dioxane has received particular attention in the recent decades since the technology is effective, cost-efficient, and environment friendly [12,13].

Until recently, a number of phylogenetically diverse microbes including bacteria and fungi that can metabolically or co-metabolically degrade 1,4-dioxane have been isolated and identified [3,19,20]. Some of these dioxane-degraders have the ability to completely mineralize 1,4-dioxane to CO_2_ [16,21,22]. Microorganisms that express monooxygenase enzymes are capable of degrading 1,4-dioxane [23]. The microbial degradation of 1,4-dioxane starts with the initial oxidation of the carbon atom adjacent to the oxygen atom known as 2-hydroxylation, which is catalyzed by soluble di-iron monooxygenases (SDIMOs) and precedes the cleavage of high energy C–O bond of the 1,4-dioxane [23,24]. This is the common rate-limiting step in the proposed 1,4-dioxane metabolic and co-metabolic degradation pathways [3]. However, the growth and degradation rates of most of the previously reported dioxane-degraders are found to be low [8]. In addition, several studies found that 1,4-dioxane biodegradation using pure strains can be inhibited in the presence of co-contaminants like heavy metals, chlorinated and fluorinated compounds, or additional carbon sources like aromatic hydrocarbons [11,24,25,26], by affecting the microbes, their metabolic activity, and the responsible enzymes [3,27,28]. On the other hand, metabolic degradation is considered to be relatively advantageous over the co-metabolic degradation, since the latter process requires additional substrates to supply auxiliary electron donors for inducing dioxane-degrading enzymes in microbial populations [29]. In these circumstances, stable microbial consortia that are capable of metabolically degrading 1,4-dioxane can be an effective solution, even in the presence of co-contaminants or additional carbon sources. The mixture of microbial species that coexist in a consortium might play an important role in the mineralization of 1,4-dioxane, whereas some microbes can initiate the 1,4-dioxane degradation and others can assist in mineralization by degrading the intermediates. Moreover, microbial consortia offer additional benefits for industrial wastewater treatment, since most of the bioreactors currently in operation are primarily based on microbial consortia [30]. However, no stable microbial consortium has been developed yet that is capable of utilizing 1,4-dioxane at different concentrations as a sole carbon and energy source.

In this study, we enriched stable 1,4-dioxane-degrading microbial consortia from dioxane contaminated industrial wastewater, which can utilize 1,4-dioxane as a sole carbon and energy source under aerobic conditions. In addition, we successfully isolated and identified two 1,4-dioxane-degrading bacterial strains, *Pseudonocardia* sp. TS06 and *Variovorax* sp. TS13 (a novel dioxane-degrader), from the consortium 112.

## 2. Materials and Methods

### 2.1. Chemicals

All chemicals and solvents used in this study were reagent-grade and were obtained from Wako Pure Chemical Industries, Ltd., Osaka, Japan.

### 2.2. Enrichment of 1,4-dioxane-Degrading Microbial Consortia

The 1,4-dioxane contaminated industrial wastewater collected from Sendai, Japan, was used as inoculum for enrichments using minimal media with 1,4-dioxane. For the enrichment of consortium 112, 3 mL of collected wastewater was added into an autoclaved 120 mL vial bottle containing 47 mL of a sterilized nitrate mineral salt medium (NMSM) supplemented with 50 mg/L of 1,4-dioxane as the sole carbon and energy source. The NMSM was composed of (per liter) 1 g of K_2_HPO_4_ and 1 g of NH_4_Cl mixed with 10 mL of mineral solution (1.5 g of nitrilotriacetic acid (NTA), 3 g of MgSO_4_·7H_2_0, 0.5 g of MnSO_4_·H_2_O, 1 g of NaCl, 0.1 g of FeSO_4_·7H_2_O, 0.1 g of CoCl_2_·6H_2_O, 0.1 g of CaCl_2_, 0.1 g of ZnSO_4_·7H_2_O, 0.01 g of CuSO_4_·5H_2_O, 0.01 g of KAl(SO_4_)_2_·12H_2_O, 0.01 g of H_3_BO_3_, and 0.01 g of Na_2_MoO_4_·2H_2_O per liter of milli-Q water) and 10 mL of vitamin solution (2 mg of biotin, 2 mg of folic acid, 10 mg of pyridoxine-HCl, 5 mg of thiamine-HCl, 5 mg of riboflavin, 5 mg of nicotinic acid, 5 mg of Ca-pantothenate, 5 mg of P-aminobenzoic acid, 5 mg of lipoic acid, and 0.1 mg of vitamin B12 per liter of milli-Q water). The pH of the medium was adjusted to 7.0 using KOH. The vial bottle was incubated at 30 °C, 170 rpm. The degradation of 1,4-dioxane was measured regularly, and 3 mL of the culture was transferred into 47 mL of NMSM, two days after the complete degradation of 50 mg/L of 1,4-dioxane was confirmed. After 4–5 successive transfers, 3 mL of the enrichment culture was then transferred into 47 mL of NMSM supplemented with 100 mg/L of 1,4-dioxane, and was incubated under similar culture conditions. This enrichment culture was considered to be the 1st generation of consortium 112. Subculturing was performed every month (after 30 days) to generate the next generation of consortium. Another consortium, namely N112, was generated from the 7th generation of the consortium 112. For consortium N112, the 7th generation of 112 was first enriched by the Luria–Bertani (LB) medium, followed by the mineral salt medium (MSM) that contained (per liter) 112 mg of MgSO_4_·H_2_O, 5 mg of ZnSO_4_·H_2_O, 2.5 mg of Na_2_MoO_4_·2H_2_O, 340 mg of KH_2_PO_4_, 670 mg of Na_2_HPO_4_·7H_2_O, 17 mg of CaSO_4_, 0.22 mg of FeSO_4_·7H_2_O, and 613 mg of (NH_4_)_2_SO_4_ [31], and was supplemented with 100 mg/L of 1,4-dioxane. The pH of the MSM was also adjusted to 7.0 and the culture condition was the same as consortium 112.

### 2.3. Analytical Methods

The concentrations of 1,4-dioxane were measured by headspace–gas-chromatography/mass spectrometry (HS–GC/MS) using a PerkinElmer Clarus 680 GC combined with a PerkinElmer Clarus SQ 8C MS (PerkinElmer, Waltham, MA, USA). The GC/MS was equipped with an EPA-624 capillary column (60 m × 0.25 mm i.d.; film thickness 1.4 µm; Restek, Bellefonte, PA, USA). Helium gas was used as the carrier gas. The column temperatures were held at 40 °C for 1 min, ramped at 10 °C/min to 200 °C, and was held at 230 °C for 3 min. The selected ion monitoring (SIM) mode was used to obtain the signals at m/z 58 and 88 for 1,4-dioxane, and m/z 64 and 96 for 1,4-dioxane-d8 as the internal standard. The detection limit was 0.001 mg/L. In brief, 2 mL of the culture sample was filtered through the membrane filter (Millex-GV, pore size: 0.22 µm, Merck Millipore, Burlington, MA, USA) and then transferred to a fresh 20 mL vial bottle containing 8 mL of milli-Q water and 3 g of NaCl. The vial bottles were then sealed with a rubber stopper and the solution was mixed thoroughly using a vortex-shaker (Vortex Genie 2-G560, Bohemia, NY, USA). Later, the sample was exposed to a water bath BM-100 (Yamato Scientific, Tokyo, Japan) for 30 min, at 70 °C. The headspace gas sample was then collected using a gastight syringe equipped with a side-sport needle, and was injected into the GC in order to measure the 1,4-dioxane concentration.

### 2.4. Degradation Experiments

To evaluate the functional stability and degradation efficiency of the consortia, different generations of consortium 112 and N112 were used to degrade 100 mg/L of 1,4-dioxane and the temporal variations in 1,4-dioxane concentration were investigated. On the other hand, to assess the effects of the initial 1,4-dioxane concentration on the degradation, 3 mL of each consortium was taken into 120 mL vial bottle containing sterilized 47 mL of MSM supplemented with different concentrations (50, 100, 500, and 1000 mg/L) of 1,4-dioxane. Control systems without microbial inoculation were also prepared. 1,4-dioxane concentrations were then measured for each bottle on the following days. All experiments were conducted in duplicates.

### 2.5. Isolation and Identification of 1,4-dioxane-Degrading Strains from Consortium 112

For the isolation of 1,4-dioxane-dgrading bacterial strains, microbial inoculum from the 30th generation of consortium 112 was streaked on NMSM mixed agar plates. After 7 days of incubation of the plates at 30 °C, single colonies of distinct bacterial species were identified and inoculated into the autoclaved 20 mL vial bottles containing 10 mL of MSM supplemented with 100 mg/L of 1,4-dioxane. The vial bottles were then incubated at 30 °C, 170 rpm, and the 1,4-dioxane concentration in each bottle was measured after 11 days of incubation to confirm if the bacterial isolates have 1,4-dioxane-degrading ability. Two isolated strains were found to be 1,4-dioxane-degraders, which were then successfully identified by sequencing the amplified 16S ribosomal RNA (rRNA) genes.

For the identification, bacterial DNA was extracted from the isolated strains using DNA Purification Kit from Wizard Genomics (Promega, Madison, WI, USA), following the manufacturer’s protocol. The 16S rRNA genes of the isolated strains were then amplified by polymerase chain reaction (PCR) analysis using PCR System 9700 (Applied Biosystems, Waltham, MA, USA) and the primer set 27F/1492R (Table 1) obtained from Eurofin Genomics Co., Ltd. The PCR was executed under the conditions: 95 °C for 2 min followed by 30 cycles of denaturation at 95 °C for 15 s, annealing at 54 °C for 20 s, elongation at 72 °C for 90 s, and finally extension at 72 °C for 7 min. Sequencing was done using a BigDye Terminator v3.1 sequencing kit and a 3130 Genetic Analyzer (Applied Biosystems, Waltham, MA, USA) and the purification was carried out using the BigDye XTerminator purification kit, after a cycle sequence reaction. The decoded base sequence was edited using the Codon Code Aligner and finally the identification was done using the GenBank database and BLAST. The phylogenetic tree was also constructed from the 16S rRNA partial sequences by using MegaX to compare with the previously reported 1,4-dioxane-degraders.

### 2.6. Microbial Community Analysis

The total DNA of the consortia was also extracted using the DNA purification kit from Wizard Genomics (Promega, Madison, WI, USA), following the manufacturer’s protocol. The microbial community analysis was done by using ribosomal RNA intergenic spacer analysis (RISA) and next generation sequencing (NGS). For RISA, the DNA extracted from the consortia was used as templates for PCR using the primer set ITSF/ITSR, which targeted the region between 16S and 23S subunits. The amplification was done by PCR analysis using PCR System 9700 (Applied Biosystems, Waltham, MA, USA) under the conditions—sample was held at 95 °C for 1 min followed by 30 cycles of denaturation at 98 °C for 10 s, annealing at 55 °C for 30 s, elongation at 72 °C for 2 min, and finally extension at 72 °C for 7 min. Then the PCR amplicons were used to run agarose gel (1.5%) electrophoresis and finally the bands were studied using UV transilluminator WUV-M20 (ATTO, Tokyo, Japan).

On the other hand, for NGS, the extracted DNA of consortium N112 was amplified at the V4 region by 1st PCR, under the following thermal profile—98 °C for 1 min followed by 25 cycles of denaturation at 94 °C for 30 s, annealing at 50 °C for 30 s, elongation at 72 °C for 30 s, and finally extension at 72 °C for 5 min. The 1^st^ PCR amplicon was then checked for its quality using agarose gel (2%) electrophoresis and finally sent to the Bioengineering Lab. Co., Ltd. (http://www.gikenbio.com) for performing 2^nd^ PCR and NGS analysis. The primers used for PCR in order to perform RISA and NGS are listed in Table 1.

### 2.7. Quantitative PCR (qPCR) Analysis

In order to study the growth of microbial consortium N112 with 1,4-dioxane degradation, qPCR analysis was done using the universal 16S rRNA primer set (Forward primer: 5ʹ-ACGGGCGGTGTGTAC-3ʹ, Reverse primer: 5ʹ-ATGGCTGTCGTCAGCT-3ʹ). The qPCR thermal cycling condition included sample holds at 95 °C for 30 s, followed by 30 cycles at 95 °C for 5 s, 55 °C for 30 s, and 72 °C for 30 s.

### 2.8. Accession Number

The partial 16S rRNA nucleotide sequences of the isolated strains *Pseudonocardia* sp. TS06 and *Variovorax* sp. TS13 were deposited to the GenBank and are available under the accession number MN715853 and MN715854, respectively.

## 3. Results

### 3.1. 1,4-dioxane Degradation Efficiency of Microbial Consortium 112

**Figure 1:**
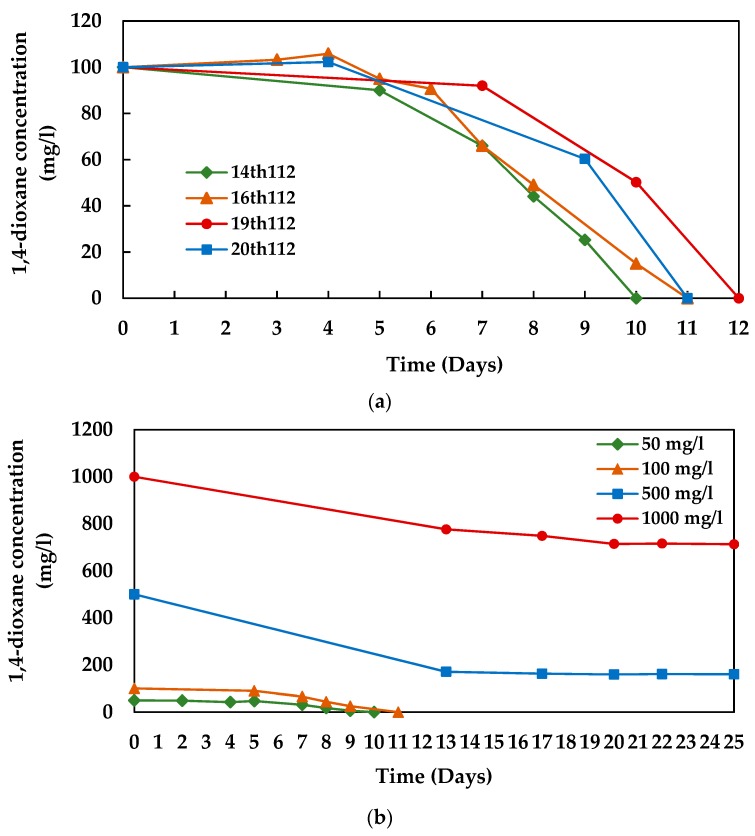
1,4-dioxane degradation profile of the consortium 112, (**a**) by different generations and (**b**) at different initial 1,4-dioxane concentrations (by 14th generation).

The degradation experiments revealed that the microbial consortium 112 enriched with NMSM was capable of utilizing 1,4-dioxane as a sole carbon and energy source. The 1,4-dioxane degradation profile of the consortium 112 is shown in Figure 1. The stable consortium 112 could completely degrade 100 mg/L of 1,4-dioxane within 10–12 days (Figure 1a). The consortium required 85 h to decrease 1,4-dioxane concentration from 80 to 20 mg/L and the degradation rate was calculated as 0.71 mg/L·h. However, an initial lag phase of 4–5 days was observed prior to the onset of 1,4-deioxane degradation by different generations of the consortium (Figure 1a), which indicated that the microbes presented with the consortium 112 required time to recover their ability and start the degradation of 1,4-dioxane.

The study observed that the enriched consortia 112 could completely degrade 1,4-dioxane at the lower concentrations (≤ 100 mg/L). The consortium 112 (14th generations) completely degraded 50 and 100 mg/L of 1,4-dioxane within 10 and 11 days, respectively (Figure 1b). Therefore, we anticipated that the consortium 112 would take a longer time to degrade higher concentrations of initial 1,4-dioxane, and subsequently observed the expected results. Though the enriched consortium 112 was found to be effective in degrading higher concentrations (500–1000 mg/L) of 1,4-dioxane, complete degradation was not obtained even after 25 days. On day 13, only 60 and 20% of the initial concentrations of 500 and 1000 mg/L of 1,4-dioxane were consumed, respectively, by the consortium 112 (Figure 1b). Afterwards, no noticeable degradation was observed for 25 days. The microbial consortium 112 degraded only 68.2% and 28.6% of the initial concentrations of 500 and 1000 mg/L of 1,4-dioxane, respectively, after 25 days of incubation (Figure 1b).

### 3.2. Isolation and Identification of 1,4-dioxane-Degrading Strains from Consortium 112

After confirming the functional stability and degradation efficiency, we tried to isolate and identify 1,4-dioxane-degrading strains from the consortium 112. Specifically, we isolated and identified two 1,4-dioxane-degrading bacterial strains, namely strain TS06 and strain TS13, from the 30th generation of the consortium 112. We observed complete degradation of 100 mg/L of 1,4-dioxane in the culture medium when we measured the 1,4-dioxane concentration after 11 days of incubation. The results, thus, confirmed that both of the isolated strains could utilize 1,4-dioxane as a sole carbon and energy source. The 16S rRNA gene sequencing analysis and BLAST search revealed that the strain TS06 belongs to the genus *Pseudonocardia*, while the strain TS13 belongs to the genus *Variovorax*. The 16S rRNA partial gene sequence of strain TS06 (*Pseudonocardia* sp.) was 99.79% (of 1374 bp) identical to the previously identified well-known 1,4-dioxane-degrader *Pseudonocardia dioxanivorans* CB1190 (Table 2), which is a gram-positive bacterium belonging to class Actinobacteria [32,33]. Whereas the 16S rRNA gene sequence of strain TS13 (*Variovorax* sp. TS13) was 99.50% (of 1,380 bp) identical to the *Variovorax paradoxus* NBRC 15149 (Table 2), which is a gram-negative bacterium belonging to class β-proteobacteria and is known to have the ability to degrade many recalcitrant organic compounds [34].

**Figure 2:**
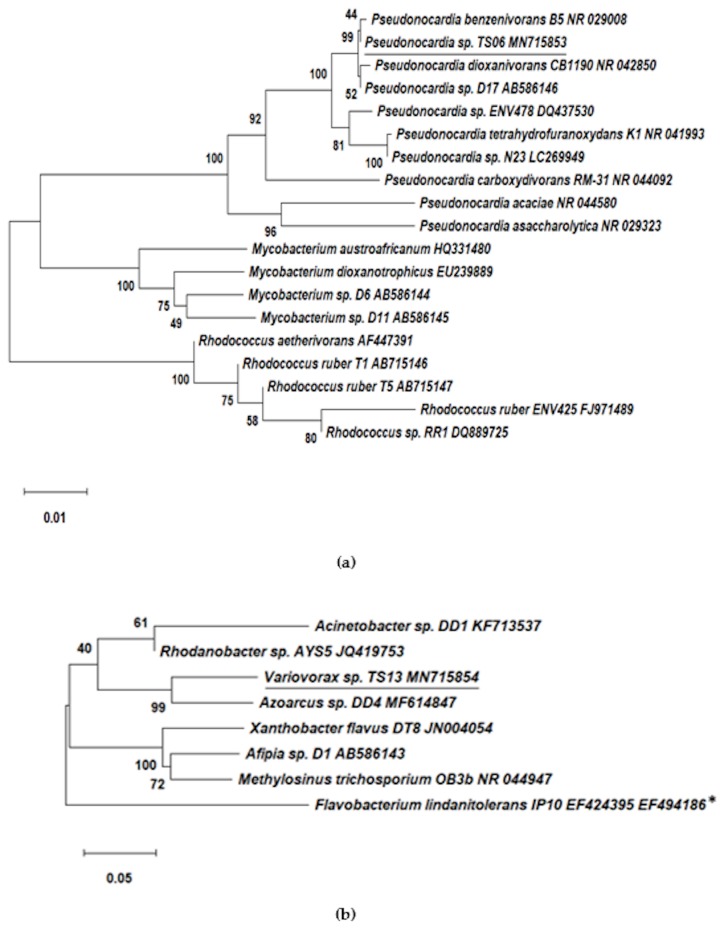
Neighbor-joining phylogenetic tree of (**a**) *Pseudonocardia* sp. TS06 (underlined) with previously reported gram-positive 1,4-dioxane-degraders and (**b**) *Variovorax* sp. TS13 (underlined), with previously identified gram-negative 1,4-dioxane-degraders. The bootstrap values were based on 1000 replications. * The *Flavobacterium lindanitolerans* IP10 was not a dioxane-degrader but was used in the analysis since it was 99% similar to 1,4-dioxane-degrader *Flavobacterium* [35].

**Figure 3:**
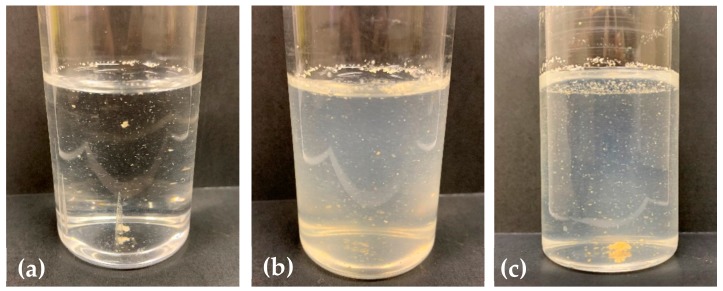
Formation of cell aggregates in liquid medium: (**a**) Consortium 112, (**b**) *Pseudonocardia* sp. TS06, and (**c**) *Variovorax* sp. TS13.

Further phylogenetic analysis showed that the *Pseudonocardia* sp. TS06 belongs to the group of previously reported 1,4-dioxane-degrading gram-positive Actinomycetes (Figure 2a). Conversely, the *Variovorax* sp. TS13 reported for the first time as a 1,4-dioxane-degrader in this study was distinct from the previously identified gram-negative dioxane-degraders (Figure 2b). Moreover, the formation of cell aggregates was observed for the isolated strains, like that in consortium 112, when cultured in a liquid medium (Figure 3).

### 3.3. 1,4-dioxane Degradation Efficiency of Consortium N112

**Figure 4:**
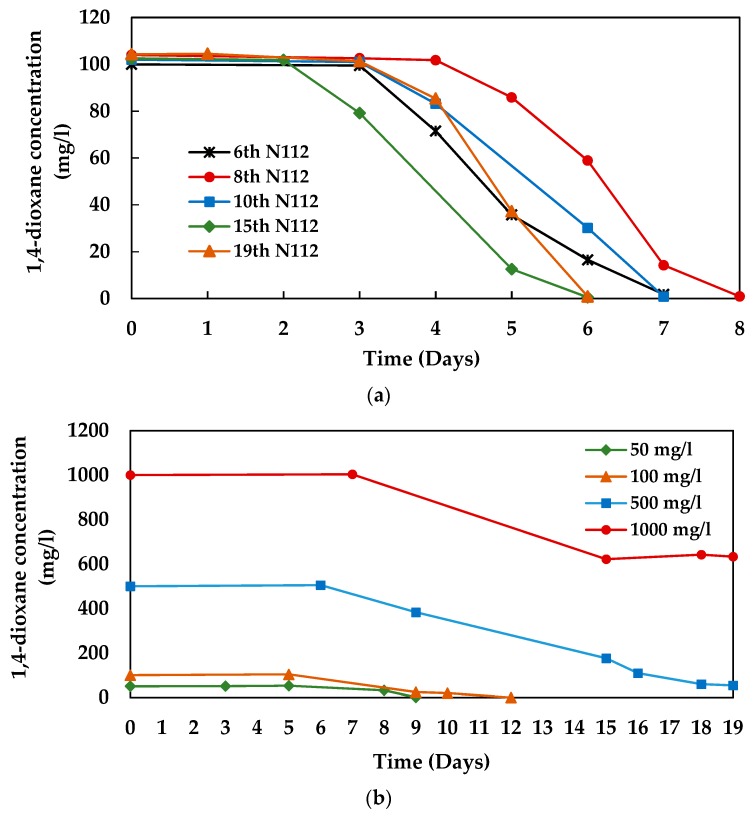
1,4-dioxane degradation profile of the consortium N112, (**a**) by different generations and (**b**) at different initial 1,4-dioxane concentrations (by 2nd generation).

From the degradation experiments, we found that the consortium N112 enriched from the consortium 112 with MSM evolved to be highly efficient in the utilization of 1,4-dioxane as a sole carbon and energy source. The 1,4-dioxane degradation profile of the consortium N112 is shown in Figure 4. The microbial consortium N112 could degrade 100 mg/L of the initial 1,4-dioxane concentration, within 6–8 days (Figure 4a). Consortium N112 required only 36 h to reduce the 1,4-dioxane concentration from 80 to 20 mg/L, which was more than one half of that obtained by consortium 112. The rate of 1,4-dioxane degradation by N112 was calculated as 1.67 mg/L·h, and the results, therefore, revealed that consortium N112 was quite capable of utilizing 1,4-dioxane as a sole carbon and energy source, and degraded 1,4-dioxane more efficiently at a rate that was 57% faster than that of consortium 112. However, like consortium 112, an initial lag phase of 2–4 days was also observed during the degradation process by different generations of N112 (Figure 4a). Moreover, we observed that consortium N112 degraded 50 and 100 mg/L of 1,4-dioxane within 9 and 12 days, respectively, and was also found to be effective in degrading higher concentrations (500–1000 mg/L) of 1,4-dioxane. Though complete degradation was not obtained (utilized only 40%) when the initial 1,4-dioxane concentration was 1000 mg/L, almost complete degradation of 500 mg/L of initial 1,4-dioxane was obtained after 19 days of incubation (Figure 4b). Interestingly, we also observed that the efficiency of consortium N112 was increased with increasing generations. For instance, the 2nd generation of N112 took 12 days to completely degrade the 100 mg/L of 1,4-dioxane (Figure 4b), while the 6th, 8th, and 10th generations took 7–8 days and the 15th and 19th generations took only 6 days to complete the job (Figure 4a). Thus, it might be possible to achieve a higher degradation efficiency if we could apply 15–19th generations of N112 instead of 2nd generation to degrade higher concentrations of initial 1,4-dioxane. Thus, the study confirmed N112 as an efficient 1,4-dioxane-degradaing microbial consortium that is capable of degrading lower to relatively higher concentrations of 1,4-dioxane within few weeks, as a sole carbon and energy source.

### 3.4. Growth of N112 with 1,4-dioxane Degradation

**Figure 5:**
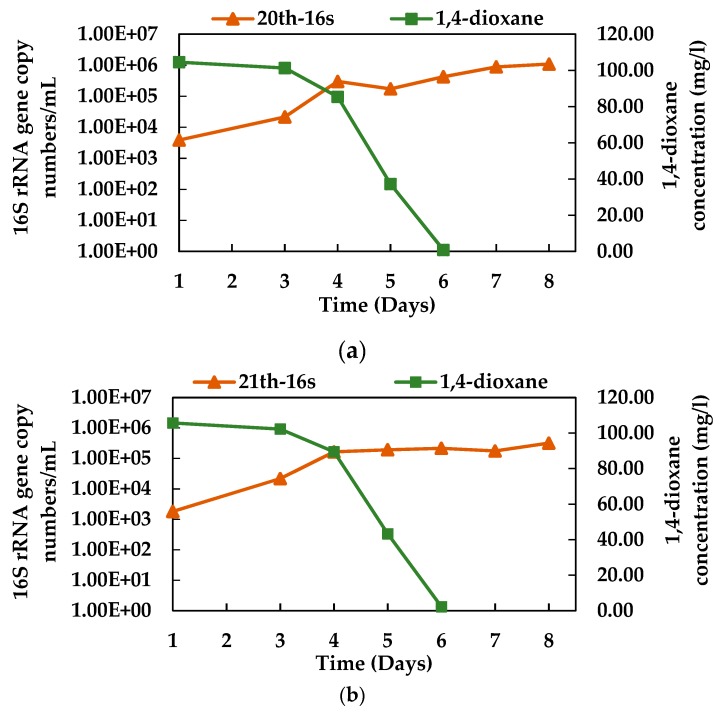
Growth of the enriched microbial consortia N112 with the degradation of 1,4-dioxane at different generations, (**a**) 20th generation and (**b**) 21st generation. Here, the rectangles indicate 1,4-dioxane concentrations and the triangles represent the overall growth of consortium N112, using 16S rRNA gene copy numbers/mL.

Figure 5 shows the 1,4-dioxane degradation profile and simultaneous growth of enriched microbial consortium N112, which was found to be the most effective as compared to the microbial consortium 112. The 20th and 21st generations of N112 were used to assess the growth of N112 with the degradation of 100 mg/L of initial 1,4-dioxane. The study found that both the 20th and 21st generations of N112 completely degraded 100 mg/L of 1,4-dioxane within 6 days, and the 16S rRNA gene copy numbers of N112/mL were increased with a decrease in 1,4-dioxane concentration (Figure 5a). After 6 days of incubation, the 16S rRNA gene copy numbers/mL were found to be increased from 3.88 × 10^3^ to 4.24 × 10^5^ for 20th generation (Figure 5a) and from 1.82 × 10^3^ to 2.16 × 10^5^ for the 21st generation (Figure 5b). Moreover, a further increase in gene copy numbers/mL was observed even after 8 days, while 1.09 × 10^6^ and 3.20 × 10^5^ gene copy numbers/mL was measured for the 20th and 21st generation, respectively.

### 3.5. Analysis of the Microbial Community Structure

**Figure 6:**
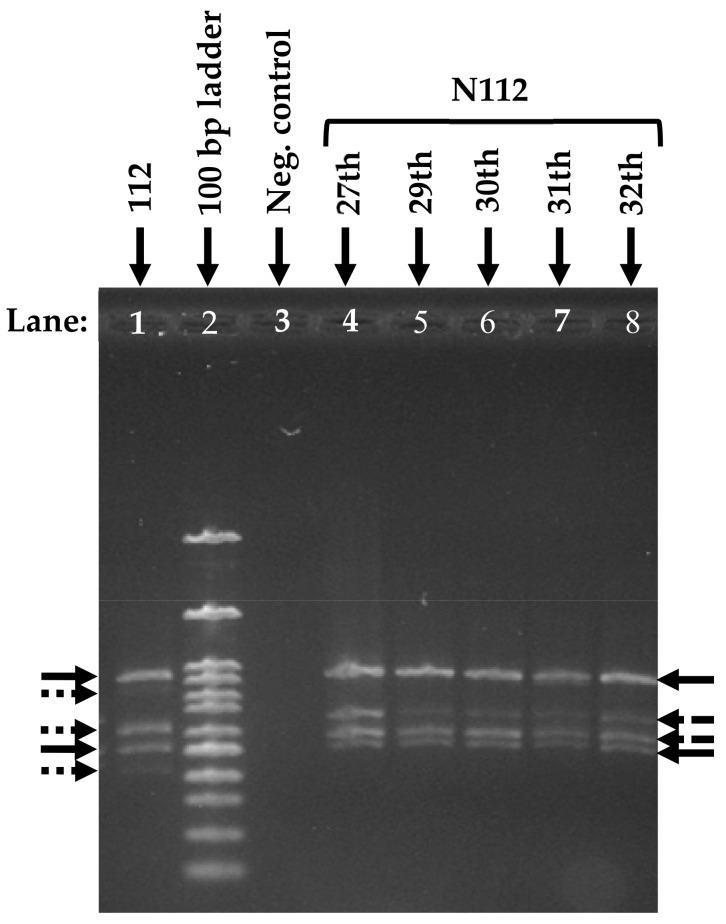
Change in the microbial community structure upon shifting from consortium 112 to N112. Here, the solid arrows represent similar bacterial species in the consortia, while the dotted (round or square) arrows represent the distinct bacterial species.

RISA was performed to investigate the microbial community structures of consortium 112 and N112. The results of RISA revealed that the microbial community structure of N112 was different from the consortium 112. The DNA bands obtained from RISA depicted that the consortium 112 was composed of five bacterial species, whereas the consortium N112 obtained from consortium 112 consisted of four bacterial species (Figure 6). The change in the microbial community structure might be the reason behind the improved 1,4-dioxane degradation ability of the consortium N112, as compared to consortium 112. However, cell aggregation was also observed for consortium N112 like that in consortium 112, which exhibited that the major 1,4-dioxane-degraders present in 112 might also be present in the consortium N112. The observed DNA bands also confirmed that consortium N112 was a stable 1,4-dioxane-degrading microbial consortium.

**Figure 7:**
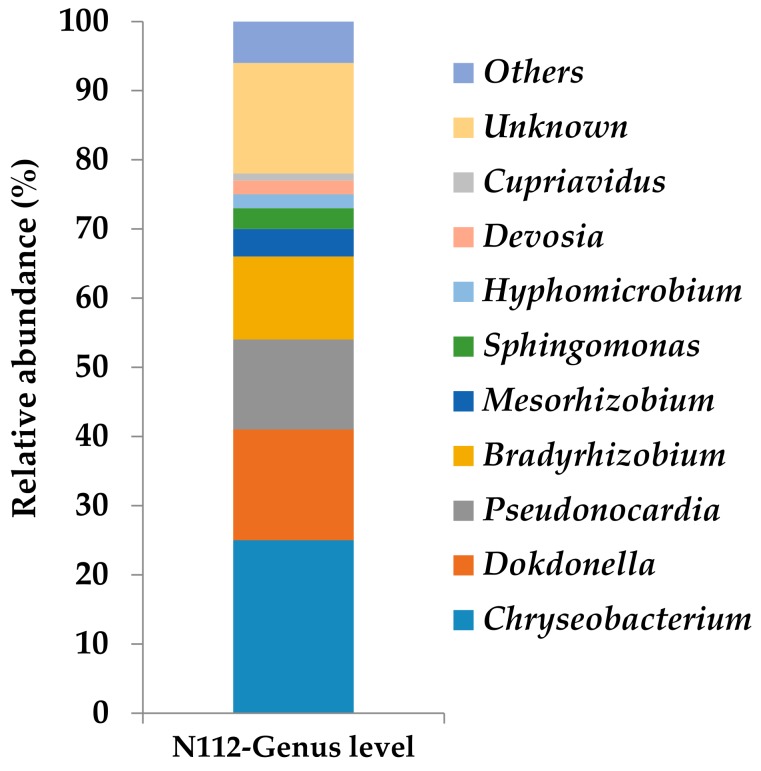
Relative abundance of bacteria at the genus level in the microbial consortium N112 (27th generation).

The results of the NGS done for consortium N112 revealed that the consortium was composed of a mixture of bacterial species belonging to the genera *Chryseobacterium* (25%), *Dokdonella* (16%), *Pseudonocardia* (13), *Bradyrhizobium* (12), *Mesorhizobium* (4%), *Sphingomonas* (3%), *Hyphomicrobium* (2%), *Devosia* (2%), and *Cupriavidus* (1%) (Figure 7). Moreover, a comparatively higher abundance of unknown members belonging to the class Saprospirae (16%) was also present in consortium N112. The genera *Pseudonocardia* and *Chryseobacterium* belong to the phyla Actinobacteria and Bacteroidetes, respectively, whereas the other genera (including the unknown group) belong to the phylum Proteobacteria. Among these bacterial populations, only the members belongings to the genus *Pseudonocaria* have been known to be potential 1,4-dioxane-degraders [32,33].

## 4. Discussion

Although significant advancements were achieved in terms of biodegradation of 1,4-dioxane using pure strains, particular attention has recently been given to the 1,4-dioxane-degrading microbial consortia composed of indigenous microbial strains as they have numerous advantages like functional stability and broader metabolic capabilities. In this study, we enriched stable microbial consortia from industrial wastewater that are capable of utilizing 1,4-dioxane at different concentrations as a sole carbon and energy source under aerobic conditions. Among the two enriched consortia, the stable consortium N112 was found as a highly effective 1,4-dioxane-degrading consortium, as compared to the consortium 112, since shorter lag phase and efficient degradation of higher 1,4-dioxane concentrations were observed by consortium N112 (Figure 4).

Apart from our study, only three studies [10,36,37] have been conducted that collectively enriched only five microbial consortia that can degrade 1,4-dioxane as a sole carbon and energy source. Arulazhagan et al. [36] enriched one bacterial consortium that can degrade 74% of 100 mg/L of 1,4-dioxane in 72 h. However, the enriched consortium was unable to completely degrade 1,4-dioxane, since no degradation was observed after 72 h [36]. Our enriched consortium N112 can degrade 1,4-dioxane at a rate of 1.67 mg/L·h, which was found to be higher than the degradation rate of a propanotroph strain SL-D (0.83 mg/L·h) developed by Innovative Engineering Solutions Inc. (IESI) [3]. However, the degradation rate of our consortia was comparatively lower than the degradation rates reported for other 1,4-dioxane-degrading pure strains, such as *Mycobacterium dioxanotrophicus* PH-06 (2.5 mg/L·h) and *Acinetobacter* sp. DD1 (2.38 mg/L·h) [3]. Since the microbial consortia are more preferable than the pure strains, our enriched stable consortium N112 has a great potential for the bioremediation of 1,4-dioxane contaminated sites and especially for the treatment of contaminated industrial wastewater.

The study also revealed that the 1,4-dioxane-degrading bacterial strains can coexist as a stable degrading enrichment and exhibit stable functionality when cultivated in the presence of 1,4-dioxane as a sole carbon and energy source. These findings are in line with the existing empirical literature. For instance, Cui et al. [38] reported that two bacterial strains capable of degrading 1,2-dichlorobenzene can coexist and show a stable degradation efficiency, if stable enrichment is maintained. Arulazhagan et al. [36] also observed five bacterial strains cohabited in the 1,4-dioxane-degradaing consortium and played a role in the degradation. However, an initial lag phase of several days was observed, prior to the onset of 1,4-dioxane degradation by both enriched consortia in our study (Figure 1a and Figure 4a). Since both consortia were maintained by subculturing them every month (after 30 days) to the next generation with 100 mg/L of initial 1,4-dioxane as a sole carbon and energy source, the 1,4-dioxane-degraders present in the consortia might become dormant after a complete utilization of 100 mg/L of the initial 1,4-dioxane. Thus, they required time (4–5 and 2–3 days for consortium 112 and N112, respectively) to recover their degradation ability after subculturing to new generations with 1,4-dioxane, which might be the possible cause of the observed lag phase during the degradation process. Nevertheless, a sharp decline in the 1,4-dioxane concentrations was observed just after the lag phase. In our future study, we are planning to perform subculture immediately after the complete degradation of 1,4-dioxane in order to maintain the consortia and also to increase the degradation efficiency by minimizing the lag phase.

In our study, we successfully isolated and identified two 1,4-dioxane-degrading bacterial strains, *Pseudonocardia* sp. TS06 and *Variovorax* sp. TS13, from the consortium 112 (Table 2). Among the isolates, the strain TS06 was found to be 99.79% identical to the 16S rRNA partial gene sequence of *Pseudonocardia dioxanivorans* CB1190, which is a rod-like bacterium and well-known 1,4-dioxane-degrader that utilizes 1,4-dioxane as a sole carbon and energy source [32,33]. The *Pseudonocardia dioxanivorans* CB1190 possesses one gene cluster (i.e., *thm*ADBC) that encodes the dioxane/tetrahydrofuran monooxygenase belonging to group 5 SDIMO enzymes that catalyze the initial hydroxylation of 1,4-dioxane by inserting a hydroxyl group at the α-carbon position of dioxane; leading to the subsequent ring cleavage of high energy C–O bond [39]. The SDIMOs, found in phylogenetically diverse bacteria, are multicomponent enzymes that are divided into six groups and play a key role in initiating bacterial oxidation of a variety of priority pollutants, including aromatic hydrocarbons, chlorinated solvents, alkanes, and alkenes [37,40]. On the other hand, the strain TS13 was the first reported bacterial species belonging to the genus *Variovorax*, which can metabolically utilize 1,4-dioxane under aerobic conditions. The identified strain TS13 was found to be 99.50% identical to the 16S rRNA partial gene sequence of *Variovorax paradoxus* NBRC 15149, which is shaped like a curved rod and forms yellow colonies in color [34]. The aggregated cells of the isolated strain *Variovorax* sp. TS13 were also observed to be yellow in color (Figure 3c).

Owing to the multiple metabolic features, the strain *Variovorax paradoxus* can survive independently and symbiotically, and can also adapt superbly in changing environmental conditions [41]. The diverse metabolic capabilities have supported the *Variovorax paradoxus* to degrade a wide range of natural and xenobiotic compounds [34]. For instance, Snellinx et al. [42] reported that the *Variovorax paradoxus* strain VM685 is capable of degrading 2,4-dinitrotoluene (2,4-DNT) and can coexist with other 2,4-DNT-degrading strain *Pseudomonas* sp. VM908. Zaitsev et al. [43] isolated *Variovorax paradoxus* strain CL-8 from the mixed culture, and reported that the strain can aerobically utilize methyl tert-butyl ether (MTBE) as a sole carbon and energy source. The possible pathway of MTBE degradation by the strain CL-8 starts with a hydroxylation of the *O*-methyl group that produces tert-butoxymethanol through the cleavage of the methyl C–H bond [43]. The MTBE has also been reported as a co-compound of 1,4-dioxane [44] and, thus, it seems possible to find the MTBE-degraders in dioxane-impacted environments where they might also play a crucial role in the degradation of 1,4-dioxane. Han et al. [41] revealed that the genome of *Variovorax paradoxus* strain S110 contains an alkane monooxygenase (*alk*B) gene, which might be the enzyme responsible for the degradation of 1,4-dioxane by the isolated strain *Variovorax* sp. TS13 in this study. Further genetic and enzymatic studies are, therefore, needed for a molecular characterization of this novel 1,4-dioxane-degrader and also for confirming the SDIMO particularly involved in the degradation.

On the other hand, although the consortium N112 was derived from consortium 112, no member belonging to the genus *Variovorax* was observed in the microbial community of consortium N112 (Figure 7). The study indicated that there is a high possibility of finding new 1,4-dioxane-degraders from consortium N112, which exhibited a higher 1,4-dioxane degradation performance and consisted of a mixture of bacterial species, of which only one genus (*Pseudonocardia*) has been known for its dioxane-degradation ability. The function of a microbial consortium is determined by its composition and is driven by the interspecies interactions [45]. Since the microbial composition of consortium N112 was found to be distinct from consortium 112, we assumed that there might be strong cooperative interactions between the species present in consortium N112. Therefore, future study is required to isolate and identify the bacterial species coexisting in consortium N112 in order to evaluate their 1,4-dioxane degradation ability and also to understand the role of bacterial coexistence in the degradation.

## 5. Conclusions

Since nowadays almost all studies focus on 1,4-dioxane degradation by pure strains, our study adds to the literature by reporting a stable and effective 1,4-dioxane-degrading microbial consortium that has immense potential to apply for bioremediation of dioxane-impacted sites and industrial wastewater contaminated with low to high concentrations of 1,4-dioxane. Moreover, for the first time, the study has reported a bacterium (strain TS13) belonging to the genus *Variovorax* that can utilize 1,4-dioxane as a sole carbon and energy source. The study supported that the microbial community composition is the basis of the function of microbial consortia, and found a higher possibility of finding other novel and phylogenetically distinct dioxane-degraders from consortium N112. In our future study, we will further investigate the microbial community of N112 by isolating both dioxane-degraders and non-degraders to reveal the role of bacterial coexistence in dioxane degradation. Furthermore, experiments will be conducted to evaluate the degradation kinetics of the isolated strains, especially *Variovorax* sp. TS13.

## Figures and Tables

**Table 1:** Sequences of primers used for PCR in this study.

Purpose	Target Gene	Primer Name	Primer Sequence (5ʹ → 3ʹ)
Isolation	16S rRNA	27F	AGAGTTTGATCCTGGCTCAG
		1492R	TACGGYTACCTTGTTACGACTT
RISA	16S-23S rRNA	ITSF ITSR	GTCGTAACAAGGTAGCCGTA GCCAAGGCATCCACC
NGS (1^st^ PCR)	16S rRNA (V4)	515F_MIX 806R_MIX	ACACTCTTTCCCTACACGACGCTCTTCCGATCT-NNNNN-GTGCCAGCMGCCGCGGTAA GTGACTGGAGTTCAGACGTGTGCTCTTCCGATCT-NNNNN-GGACTACHVGGGTWTCTAAT

**Table 2:** Identification of 1,4-dioxane-degrading bacterial strains and their closest relatives.

Isolated Strains	Family (Class)	Closest Relative (Accession No.)	Similarity with the Closest Relative (%)	Accession No.
*Pseudonocardia* sp. TS06	Pseudonocardiaceae (Actinobacteria)	*Pseudonocardia dioxanivorans* CB1190 (NR_074465)	99.79	MN715853
*Variovorax* sp. TS13	Comamonadaceae (β-proteobacteria)	*Variovorax paradoxus* NBRC 15149 (NR_113736)	99.50	MN715854
